# A Randomized Trial of Topical Fibrinogen-Depleted Human Platelet Lysate Treatment of Dry Eye Secondary to Chronic Graft-versus-Host Disease

**DOI:** 10.1016/j.xops.2022.100176

**Published:** 2022-06-02

**Authors:** Alan Sugar, Munira Hussain, Winston Chamberlain, Reza Dana, David Patrick Kelly, Christopher Ta, John Irvine, Melissa Daluvoy, Victor Perez, Joshua Olson, Vishal Jhanji, Terence A. Walts, Robert Doyle Stulting, Edmund K. Waller, Neera Jagirdar, Cambium Dry Eye Study Group, Alan Sugar, Shahzad Mian, Roni Shtein, H. Kaz Soong, Munira Hussain, Winston Chamberlain, Afshan Nanji, John Clements, Jennifer Maykovski, Paula Cisternas Labadzinzki, Reza Dana, Jia Jin, Joseph Ciolino, John Caccaviello, D. Patrick Kelly, Roya Habibi, Christopher Ta, Charles Yu, Charles Lin, Kristin Hirabayashi, Gabriel Valerio, Supriya Kawale, Mariana Nunez, John Irvine, Olivia Lee, Matthew Chu, Melissa Daluvoy, Victor Perez, Elmer Balajonda, Terry Hawks, Joshua Olson, Amanda Maltry, Joshua Hou, Wendy Elasky, Vishal Jhanji, Rose Carla Aubourg, R. Doyle Stulting, Edmund Waller, Neera Jagirdar, Terence Walts

**Affiliations:** 1 WK Kellogg Eye Center, University of Michigan, Ann Arbor, Michigan; 2 Casey Eye Institute, and Department of Ophthalmology, Oregon Health and Science University, Portland, Oregon; 3 Department of Ophthalmology, Massachusetts Eye and Ear Infirmary, Harvard Medical School, Boston, Massachusetts; 4 Eye Associates Northwest PC, Seattle, Washington; 5 Department of Ophthalmology, School of Medicine, Stanford University, Stanford, California; 6 Doheny Eye Center, UCLA, Los Angeles, California; 7 Duke University Eye Center, Durham, North Carolina; 8 Department of Ophthalmology and Visual Neurosciences, University of Minnesota, Minneapolis, Minnesota; 9 UPMC Eye Center, University of Pittsburgh School of Medicine, Pittsburgh, Pennsylvania; 10 Cambium Medical Technologies LLC, Atlanta, Georgia; 11 Woolfson Eye Institute, Atlanta, Georgia; 12 Winship Cancer Institute of Emory University, Atlanta, Georgia

**Keywords:** Dry eye disease, Graft versus host disease, Human platelet, Ocular surface, Serum tears, AE, adverse event, ASTs, autologous serum tears, CI, confidence interval, DED, dry eye disease, FD hPL, fibrinogen-depleted human platelet lysate, GvHD, graft-versus-host disease, HSCT, hematopoietic stem cell transplant, OSDI, ocular surface disease index, PRP, platelet-rich plasma, SAE, severe adverse event, VAS, Visual Analog Scale

## Abstract

**Purpose:**

The purpose of the study was to evaluate, as a pilot trial, safety and tolerability of CAM-101 10% and 30% topical ophthalmic fibrinogen-depleted human platelet lysate (FD hPL) solution in patients with dry eye disease (DED) secondary to graft-versus-host disease (GvHD) after 6 weeks of treatment.

**Design:**

A phase I/II, pilot, prospective, multicenter, randomized, double-masked clinical trial.

**Participants:**

Patients with DED secondary to GvHD.

**Methods:**

Sixty-four adult patients were stratified by “symptom severity” (Ocular Surface Disease Index [OSDI], ocular discomfort Visual Analog Scale (VAS), ocular symptom frequency, and use of artificial tears) and then randomized 1:1:1 to CAM-101 (FD hPL) at 10% or 30% concentration or an electrolyte (Plasma-Lyte A) vehicle control, 1 drop in both eyes, 4 times daily, for 42 days. After 42 days, control patients were offered 42 days of open-label treatment with 30% FD hPL.

**Main Outcome Measures:**

Primary outcome safety measures were ocular and systemic adverse events and the number of patients in each group with clinically significant change from normal to abnormal in any ocular findings. Secondary outcomes were changes from baseline to day 42 in ocular discomfort, OSDI, fluorescein corneal staining, and lissamine green conjunctival staining relative to the vehicle control. The ocular symptom frequency was assessed on a 100-point VAS.

**Results:**

FD hPL 10% and 30% were safe and well tolerated. Relative to the vehicle control, significant decreases from baseline to day 42 were seen in the FD hPL 30% group with regard to ocular discomfort (mean decrease = −18.04; *P* = 0.018), frequency of burning/stinging (−20.23; *P* = 0.022), eye discomfort (−32.97; *P* < 0.001), eye dryness (−21.61; *P* = 0.020), pain (−15.12; *P* = 0.044), photophobia (−24.33; *P* = 0.0125), and grittiness (−20.08; *P* = 0.0185). Decreases were also seen for itching and foreign body sensation, though not statistically significant. Improvements were seen in tear breakup time (mean increase = 1.30 seconds; *P* = 0.082) and the investigator’s global evaluation 4-point scale (mean decrease = −0.86; *P* = 0.026). Corneal fluorescein staining was not improved. The OSDI had a mean decrease of −8.88 compared to the vehicle, although not statistically significant.

**Conclusions:**

Fibrinogen-depleted human platelet lysate appears to be well tolerated, with no significant toxicity at concentrations of 10% and 30%. These initial data suggest some efficacy, especially for subjective outcome measures relative to baseline assessments and treatment with the vehicle, but larger studies are needed to confirm these effects.

Dry eye disease (DED)^1^ is a major feature of chronic graft-versus-host disease (GvHD). GvHD is a complication of hematopoietic stem cell transplantation (HSCT), which is used to treat a variety of neoplastic and genetic diseases. It is caused by immunologic activity of allogeneic donor cells against recipient tissues and occurs in up to 70% of HSCT recipients. A major feature is inflammation of mucosal surfaces, including those of the eye. A diagnosis of chronic ocular GvHD is based on symptoms (measured by the Ocular Surface Disease Index [OSDI]), Schirmer testing, corneal staining, and conjunctival inflammation.^2^

Ocular GvHD is a particularly severe form of DED that develops in 60% to 90% of patients after allogeneic HSCT.^3^ Patients with ocular GvHD may develop conjunctival scarring, keratinization and cicatrization, and corneal neovascularization with significant loss of vision.^4^ Meibomian gland dysfunction is frequently present.^5^ A decrease in tear production is observed in patients with systemic GvHD.^4^ Systemic immunosuppressive drug treatment, the primary intervention in the management of patients with chronic GvHD, may relieve ocular symptoms; however, it is often ineffective or not indicated based solely on ocular GvHD symptoms.^4^

There are currently no US Food and Drug Administration-approved treatments for ocular GvHD. Treatment of DED in GvHD usually begins with topical preservative-free tear substitutes followed by cyclosporine (Restasis, Allergan, Irvine, CA; Cequa, Sun Ophthalmics, Princeton, NJ) or lifitegrast (Xiidra, Novartis, Basel, Switzerland).^6^^,^^7^ Topical steroids may be helpful during exacerbations, though the low-dose agents often used for DED have limited efficacy.^8^ Punctal occlusion and treatment of associated meibomian gland dysfunction are also helpful. Scleral contact lenses may be used to improve both comfort and vision.^9^

Autologous serum tear (AST) drops at 20% to 50% concentration are often very effective.^10^ Autologous serum tears are thought to be helpful both because of their physical properties and the presence of growth factors, such as epidermal growth factor, platelet-derived growth factor, and fibroblast growth factor, and other factors and vitamins which promote surface healing.^11^ Other serum components, however, may promote inflammation.

Platelet-rich plasma (PRP) has been used as a means of enhancing these factors in an artificial tear, but use of plasma requires the addition of anticoagulants.^11^ Autologous platelet lysate has been shown to be useful in a small series of ocular GvHD cases; however, its preparation is difficult and time consuming, as the process involves the separate preparation of platelet lysate for each patient.^12^ To address several of the inherent shortcomings of autologous blood-derived agents, Cambium Medical Technologies LLC (Atlanta, GA) developed a method of preparing fibrinogen-depleted human platelet lysate (FD hPL) from pooled human platelet apheresis products obtained from qualified healthy donors collected at certified blood collection centers. Cambium’s FD hPL, trademarked Aurarix, is the main active ingredient, composed of platelet-derived growth factors and vitamins, of the investigational product used in the trial (CAM-101, trademarked Elate Ocular).

The present randomized phase I/II pilot study was designed to evaluate the tolerability and safety of this agent and to explore its efficacy as a proof-of-concept clinical trial and to generate hypotheses relevant to future clinical development. This pilot trial was done in ocular GvHD patients to treat that disease but also as a demanding test of dry eye therapy with FD hPL drops.

## Methods

### Study Design

**Figure 1:**
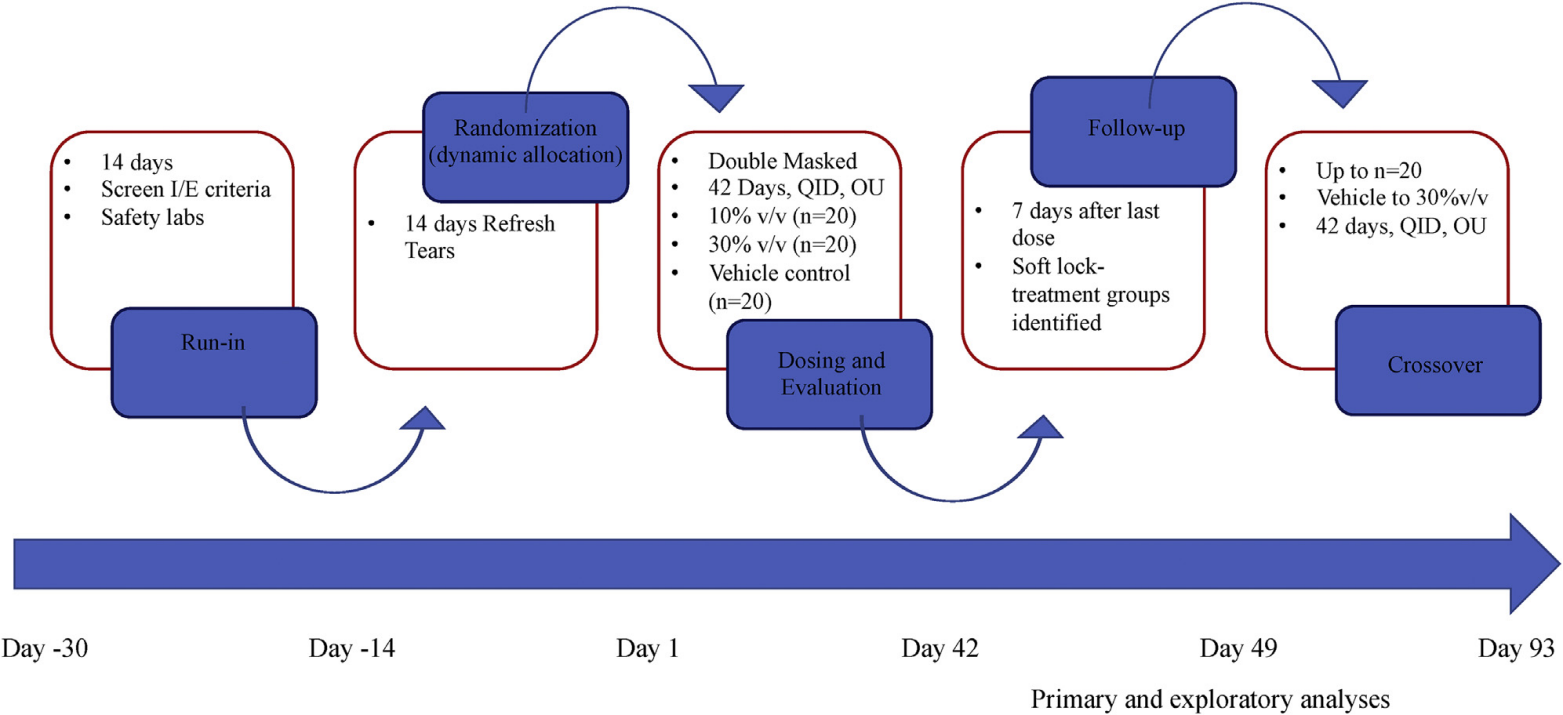
Schematic overview of the CAM-101 trial. OU = both eyes; QID = four times daily.

The CAM-101 study was a prospective, multicenter, patient-, physician-, and evaluator-masked, randomized, phase I/II pilot clinical trial. Sixty-four participants were enrolled from 9 centers throughout the United States. Institutional review board approval was obtained at each center (Massachusetts Eye and Ear Infirmary – Harvard University IRB; Oregon Health and Science University IRB; University of Michigan IRB; Duke University IRB; Stanford University IRB; University of Minnesota IRB; Eye Associates NW, Seattle – Advarra IRB; University of Pittsburgh IRB; UCLA Dept of Ophthalmology IRB), and the trial was carried out under a US Food and Drug Administration Investigational New Drug application (IND 017669). Procedures were conducted under International Council for Harmonisation–Good Clinical Practice standards, and the trial was registered with ClinicalTrials.gov (NCT 03414645). The study adhered to the principles of the Declaration of Helsinki, and informed consent was obtained from each participant. A schematic representation of the study design is presented in Figure 1.

The original inclusion criteria for the study included a prior diagnosis of DED secondary to GvHD after HSCT, a Schirmer test with anesthesia < 7 mm/5 minutes, and a tear film osmolarity > 312 mOsm/L. The tear film osmolarity criterion was eliminated early in the study when osmolarity could not be measured for several potential subjects because of inadequate or no tear volume. Subjects had to be on stable doses of systemic immunosuppressive drugs for ≥ 4 weeks.

After written informed consent was obtained, potential subjects were screened for study eligibility. After the initial screening, there was a 2-week washout period during which patients were asked to use and record the use of preservative-free artificial tears (Refresh Plus, carboxymethyl cellulose, 0.5%; Allergan) as needed. The use of topical cyclosporine (Restasis) or lifitegrast (Xiidra) was continued if patients were already taking these medications prior to enrollment. The use of other topical medications was not allowed. The continued use of punctal plugs was allowed during the run-in period if patients had punctal plugs in place at the time of enrollment. Other DED treatments (e.g., autologous serum, PRP serum) were not allowed during the study. Patients recorded the frequency of their use of artificial tears during the run-in period.

At the end of the run-in period, patients were evaluated by “symptom severity” (a composite of the OSDI score, ocular discomfort on a Visual Analog Scale [VAS], ocular symptom frequency VAS, and frequency of use for artificial tears) and subsequently randomized on day 1 on a 1:1:1 basis to 1 of 3 treatment groups. Randomization was blocked by the site based on the symptom severity score using a central interactive web response system. The treatment groups were as follows: (1) FD hPL 10 vol/vol% concentration (CAM 101 10%), (2) FD hPL 30 vol/vol% (CAM 101 30%), and (3) vehicle control (Plasma-Lyte A, a multiple electrolyte solution, pH 7.4, Baxter Pharmaceuticals).

**Figure 2:**
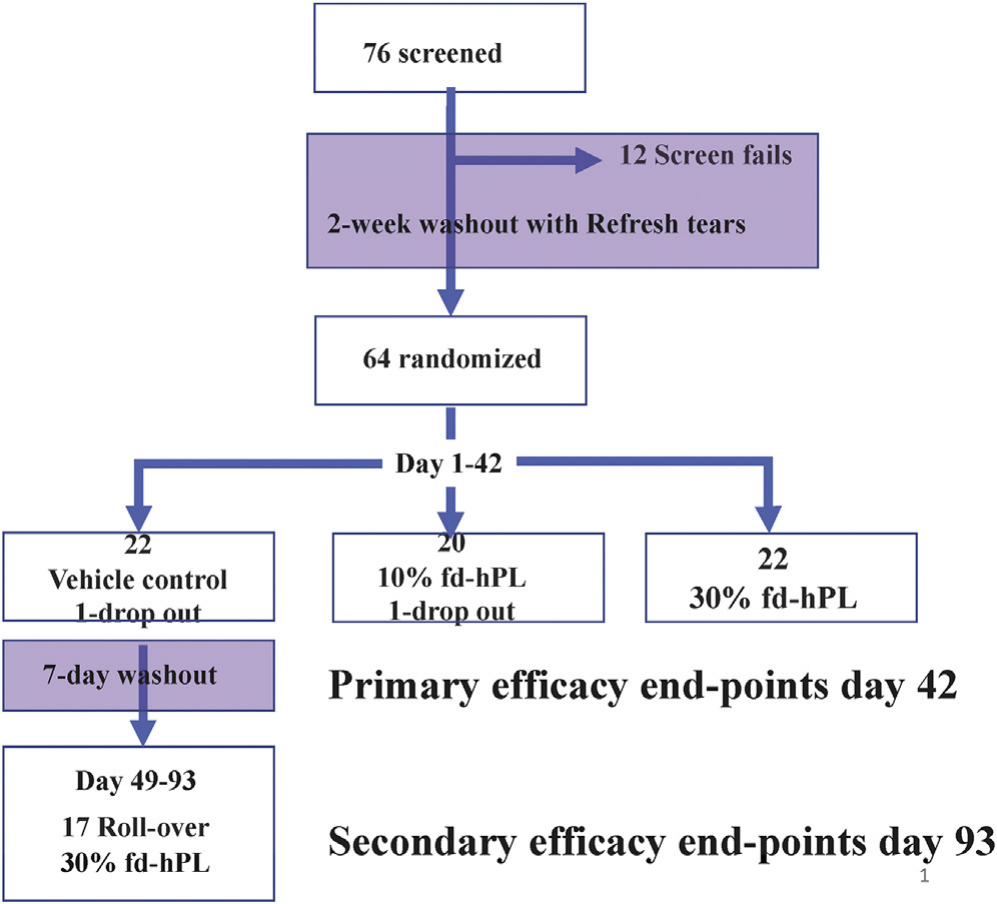
Consolidated Standards of Reporting Trials (CONSORT) diagram of the CAM-101 trial. FD hPL = fibrinogen-depleted human platelet lysate.

In addition, we had planned for a special population of highly responsive patients who experienced a significant improvement in symptom severity, subjectively defined by the investigator as having > 50% improvement in the composite of symptom severity scores, at the baseline visit relative to screening visit to be enrolled directly into the CAM 101 30% group and followed for 42 days for safety analysis. No patients were enrolled into this cohort because there were no patients who had > 50% improvement in subjective composite scores at the end of the run-in period (Fig 2).

The study agent (FD hPL) was prepared from platelet apheresis products collected from donors screened for risk of transmissible infectious diseases by the questionnaire and serial nucleic acid testing using US Food and Drug Administration-approved assays. Outdated platelet products were sourced from blood banks and frozen. The study agent was prepared from frozen-thawed pooled platelet lysates by a proprietary process involving depletion of fibrinogen. The study agent (FD hPL) was then diluted to concentrations of 10% and 30% (volume/volume) in the Plasma-Lyte A vehicle. Study groups were masked to subjects and examiners, and dropper bottles were indistinguishable. The investigational product was handled, stored, and dispensed to patients frozen. Patients were instructed to store the investigational product frozen at −20° ± 3°C (typical home freezer temperature) and to thaw the next day’s dosage (4 bottles) overnight, the day before use in their refrigerator or during the day of use and to keep the remaining product frozen until the day before use.

After baseline assessments, subjects self-administered a single study drop in both eyes and were monitored for 30 minutes for immediate side effects. Subjects were instructed to apply the study drops 4 times daily at home. Participants returned for study visits at day 21 ± 3 (visit 2) and day 42 ± 3 (visit 3) and a single follow-up visit within 7 days of the last dose at visit 4 (days 46–52). Patients reported the frequency of use of Refresh Plus (Allergan) artificial tears and other concomitant therapies at 21- and 42-day study visits.

After the follow-up visit and in the absence of any drug-related serious adverse events (AEs) during the study, patients assigned to the vehicle control group were offered the opportunity to receive open-label treatment with FD hPL 30% for 42 days.

### Outcome Measures

The primary outcome measures were based on safety assessments, including the proportion of patients with ocular and systemic AEs by day 42, and the proportion of patients in each dose group who had developed abnormal clinically significant findings during ocular examination at day 42 when their baseline findings were normal.

Secondary outcome measures were efficacy endpoints, including reductions in ocular discomfort from baseline as measured with a VAS and the OSDI, fluorescein corneal staining, and lissamine green conjunctival staining at day 42. Staining was measured using the Oxford Scale.^13^ Exploratory efficacy outcome measures included changes from baseline to day 42 in tear osmolarity, Schirmer test, and investigator’s global evaluation.

### Statistical Analysis

As this was a pilot phase I/II study, the sample size was based upon practical considerations, to provide data on possible toxicities and to reveal possible efficacy signals across a variety of measured signs and symptoms that could then be used to plan phase II/III studies. As a first-in-human study of allogeneic pooled platelet lysate drops, there were no *a priori* statistical assumptions for efficacy measures. Analyses were performed for the full subject data set and for subjects completing the treatments and assessments according to the protocol. The means, mean differences, confidence intervals (CIs), and *P* values were derived from a repeated measures model with treatment group, baseline severity category, visit, and treatment-by-visit interaction as fixed categorical effects using an unstructured covariance matrix. The CIs and *P* values were for mean changes from baseline, and *P* values were 2-tailed. Corrections for the multiplicity of analyses were performed using the Bonferroni method and did not change conclusions. SAS, version 9.3 or higher, was used as the statistical software package for all data analyses. For the analysis of the efficacy endpoints, 2-sided 95% CIs were presented for the difference in mean change from baseline between the active doses and the vehicle control. These CIs were based on a repeated measures model with treatment group, baseline severity category, visit, and treatment-by-visit as fixed factors. The modeling was done using the PROC MIXED procedure of the SAS software. The correlation among the measurements across visits was assumed to be unstructured, and a Kenward-Roger option for calculating the denominator degrees of freedom was used.^14^ Categorical endpoints were summarized using the patient count and percentage in each category with 95% CIs calculated using the Wald method.

## Results

Seventy-six subjects consented and enrolled between May 2018 and December 2019. Sixty-four subjects were randomized, including 20 subjects assigned to the FD hPL 10% group (group 1), 22 assigned to FD hPL 30% group (group 2), and 22 assigned to the control group (group 3). Of the 22 participants in the control group, 17 subsequently entered the open-label rollover phase (Fig 2). Of the 64 participants randomized in the trial, 54 (84%) completed the study according to the protocol, and 10 (16%) prematurely discontinued. The primary reason for withdrawal from the study was a change in systemic medications indicated by a change in the underlying systemic GvHD. Of the 12 who were deemed screen failures, those reasons included not meeting tear osmolarity criteria, not being able to comply with investigational product storage conditions, and chronic GvHD flare-ups requiring adjustments in systemic medications (Fig 2). Baseline demographic characteristics of the subjects in the 3 groups were comparable (Table 1). The mean age of enrolled subjects was 53 (±13.8, range = 18–77) years. Of the total participants, 36 (56%) were male and 9 (14%) self-described as belonging to a racial category other than White. The frequencies of self-reported race and ethnicities of the enrolled subjects were similar to data for allogeneic transplant recipients reported to the Center for International Blood and Marrow Transplant Research.^15^ Treatment noncompliance was defined as <80% or >120% of the expected dose as determined by the number of empty bottles returned and the patient diary. However, since not all patient diaries were available, the determination of compliance was assessed for some subjects by the number of empty bottles recorded in the compliance log. Overall, in the double-masked treatment phase, 54 (84%) of 64 patients were compliant, with 16 (73%), 19 (95%), and 19 (86%) patients in the vehicle control, FD hPL 10%, and FD hPL 30% groups, respectively, determined to be compliant. The final study visit was in February 2020.

**Table 1:** Baseline Demographic Characteristics of Participants by Treatment Group

Characteristic	Vehicle Control (N = 22)	FD hPL 10% (N = 20)	FD hPL 30% (N = 22)	Total (N = 64)
Age (years) mean (±SD)	53.1 (±13.8)	53.6 (±14.9)	53.7 (±13.5)	53.4 (±13.8)
Sex				
Male	11 (50%)	13 (65%)	12 (54.5%)	36 (56%)
Female	11 (50%)	7 (35%)	10 (45.5%)	28 (44%)
Ethnicity				
Hispanic or Latino	3 (14%)	5 (25%)	3 (14%)	11 (17%)
Non-Hispanic or Non-Latino	18 (82%)	15 (75%)	19 (86%)	52 (81%)
Not reported	1 (4%)	0	0	1 (2%)
Race				
American Indian or Alaska native	0	0	0	0
Asian	0	1 (5%)	1 (4.5%)	2 (3%)
Black or African American	1 (4.5%)	2 (10%)	0	3 (5%)
White	20 (91%)	15 (75%)	20 (91%)	55 (86%)
Other/unknown	1 (4.5%)	2 (10%)	1 (4.5%)	4 (6%)

FD hPL = fibrinogen-depleted human platelet lysate; SD = standard deviation.

Table 2 lists the baseline ocular assessments by treatment groups. Overall, baseline ocular assessments were indicative of more severe dry eye symptoms in the FD hPL 30% group than in either the vehicle control or FD hPL 10% group at baseline, but the differences were not statistically significant. All dry eye symptoms assessed, except for itching and pain, were more frequent in the FD hPL 30% group than those in the vehicle control or FD hPL 10% group, but the differences were not statistically significant. Outcomes and conclusions did not differ between intent-to-treat and per protocol analyses.

**Table 2:** Baseline Ocular Assessments by Treatment Group

Assessment	Vehicle Control (N = 22)	FD hPL 10% (N = 20)	FD hPL 30% (N = 22)
Artificial tear use	22 (100%)	20 (100%)	22 (100%)
Symptom frequency (mean ± SD)			
Burning/stinging	48.3 (±27.9)	44.9 (±33.8)	50.8 (±37.1)
Eye discomfort	61.5 (±27.9)	60.2 (±29.6)	73.4 (±27.5)
Eye dryness	76.9 (±24.1)	74.4 (±25.4)	77.5 (±28.3)
Foreign body	36.5 (±31.5)	44.3 (±31.5)	46.5 (±34.4)
Grittiness	41.9 (±33.4)	36.0 (35.05)	47.7 (32.87)
Itching	47.3 (31.37)	30.1 (24.81)	44.1 (31.51)
Pain	39.4 (29.67)	36.8 (30.35)	37.6 (34.88)
Photophobia	49.5 (31.12)	54.5 (31.72)	71.6 (35.97)
Ocular discomfort	61.4 (20.69)	60.5 (24.53)	70.3 (25.09)
Fluorescein sodium staining	3.6 (2.75)	4.3 (2.15)	4.0 (1.90)
Total lissamine green staining	9.8 (5.83)	10.2 (6.64)	12.1 (4.20)
Tear osmolarity	294.62 (23.948)	292.61 (40.033)	314.13 (21.273)
Schirmer’s test	3.16 (3.057)	3.43 (4.482)	2.95 (2.355)
Investigator’s global examination	4.41 (1.008)	4.85 (0.366)	4.57 (0.904)

AE = adverse event; m = number of events; N = number of patients in a specific group; n = number of patients with a particular AE; calculation of percentages based on N.

Treatment-emergent AEs are defined as AEs that increased in severity or newly developed after first dosing.

FD hPL = fibrinogen-depleted human platelet lysate; SD = standard deviation.

Eye irritation following study drug administration occurred in 3 of 22 controls and in 1 of 20 in 10% and 0 of 20 in 30% groups. Eye pain was also greatest in the control group (2/22, 1/20, and 0/22 for control, 10%, and 30% groups, respectively, Table 3). No severe AEs (SAEs) were attributable to study treatments. Two patients, 1 each in the vehicle control and FD hPL 10% groups, discontinued from the study due to AEs. One subject in the vehicle control group discontinued due to hospitalization secondary to underlying GvHD. In the 10% group, 1 patient was discontinued after hospitalization for sinus infection. This patient was immunocompromised due to systemic GvHD medications and had been experiencing sinus symptoms for several months before study entry. There were no ocular AEs in either the 10% or 30% group rated > 1 (mild) on a 1 to 5 scale. Overall, 7 patients in the double-masked treatment phase experienced ≥ 1 SAE, including 9% (2/22), 10% (2/20), and 14% (3/22) in the vehicle control, FD hPL 10%, and FD hPL 30% groups, respectively. No specific SAE occurred in > 1 patient. SAEs reported among FD hPL–treated patients included orbital cellulitis and febrile neutropenia in the FD hPL 10% group and parainfluenza viral pneumonia and hypertrophic cardiomyopathy (in the same patient), fungal pneumonia, and cardiopulmonary failure in the FD hPL 30% group. SAEs in the vehicle control group included respiratory syncytial virus infection and dysphagia. All SAEs were considered by the investigator to be unrelated to the study drug (Table 3).

**Table 3:** Overall Summary of Treatment-Emergent Adverse Events by Treatment Group in the Double-Masked Treatment Phase

Patients With ≥ 1:	Vehicle Control (N = 22) n (%) m	FD hPL 10% (N = 20) n (%) m	FD hPL 30% (N = 22) n (%) m
AE	10 (45.5) 34	9 (45.0) 12	8 (36.4) 11
Treatment-related AEs	1 (4.5) 2	1 (5.0) 1	0
Ocular AE	7 (31.8) 19	4 (20.0) 6	2 (9.1) 2
Treatment-related ocular AEs	1 (4.5) 1	1 (5.0) 1	0
Serious AE	2 (9.1) 2	2 (10.0) 2	3 (13.6) 4
AEs leading to study withdrawal	1 (4.5) 6	1 (5.0) 1	0

AE = adverse event; m = number of events; N = number of patients in a specific group; n = number of patients with a particular AE; calculation of percentages based on N.

Treatment-emergent AEs are defined as AEs that increased in severity or newly developed after first dosing.

FD hPL = fibrinogen-depleted human platelet lysate; SD = standard deviation.

The ocular symptom frequency was assessed by patients on a VAS of 0 to 100, with 0 indicating a frequency of “never” and 100 indicating a frequency of “constant.” Relative to the vehicle control, significant decreases in ocular symptoms from baseline to day 42 were seen in the FD hPL 30% group (FD hPL 30% minus vehicle control). Specifically, lower frequencies of burning/stinging (mean decrease = −20.23; *P* = 0.045), eye discomfort (mean decrease = −32.97; *P* < 0.001), eye dryness (mean decrease = −21.61; *P* = 0.040), pain (mean decrease = −15.12.0; *P* = 0.089), photophobia (mean decrease = −24.33; *P* = 0.025), and grittiness (mean decrease = −20.08; *P* = 0.037) were seen in subjects treated with 30% FD hPL (Table 4). Decreases from baseline to day 42 were seen in this group for itching and foreign body sensation; however, the difference between groups did not reach statistical significance. Improvements were also seen in tear breakup time (mean increase = 1.03; *P* = 0.082), ocular discomfort (mean decrease = −18.04; *P* = 0.036), and the investigator’s global evaluation (mean decrease = 0.86; *P* = 0.052) (Table 5). Although not statistically significant, the OSDI had a mean decrease of 8.88 relative to control (19.71 decrease from baseline).

**Table 4:** Ocular Symptom Frequency VAS: Comparison of Change from Baseline to Day 42 Between Treatments in the Double-Masked Treatment Phase

Parameter	Statistic	Vehicle Control (N = 22)	FD hPL 10% (N = 20)	FD hPL 30% (N = 22)	FD hPL 10%–Vehicle Control	FD hPL 30%-Vehicle Control
Burning/stinging	Mean	−6.39	−4.98	−26.62	1.41	−20.23
	95% CI	(−25.24, 12.46)	(−22.61, 12.66)	(−45.77, −7.46)	(−18.68, 21.51)	(−39.97, −0.48)
	*P* value†				**0.888**	**0.045**
Itching	Mean	−12.31	−7.18	−21.67	5.13	−9.36
	95% CI	(−28.83, 4.21)	(−22.31, 7.96)	(−38.43, −4.91)	(−11.30, 21.57)	(−25.48, 6.76)
	*P* value†				**0.534**	**0.250**
Foreign body	Mean	6.45	−10.23	−4.61	−16.68	−11.05
	95% CI	(−12.53, 25.43)	(−27.90, 7.43)	(−23.88, 14.67)	(−36.59, 3.22)	(−30.61, 8.50)
	*P* value†				**0.099**	**0.262**
Eye discomfort	Mean	2.77	−13.19	−30.20	−15.96	−32.97
	95% CI	(−15.10, 20.65)	(−29.63, 3.25)	(−48.34, −12.06)	(−33.90, 1.97)	(−50.58, −15.37)
	*P* value†				**0.080**	**<0.001**
Eye dryness	Mean	−4.32	−14.17	−25.92	−9.86	−21.61
	95% CI	(−25.37, 16.74)	(−33.49, 5.15)	(−47.29, −4.55)	(−30.86, 11.15)	(−42.22, −0.99)
	*P* value†				**0.352**	**0.040**
Photophobia	Mean	−11.20	−20.49	−35.53	−9.30	−24.33
	95% CI	(−33.73, 11.34)	(−40.98, −0.00)	(−58.38, −12.67)	(−30.95, 12.36)	(−45.57, −3.09)
	*P* value†				**0.394**	**0.025**
Pain	Mean	−1.86	−6.48	−16.97	−4.63	−15.12
	95% CI	(−19.13, 15.42)	(−22.49, 9.53)	(−34.51, 0.57)	(−22.43, 13.18)	(−32.61, 2.37)
	*P* value†				**0.605**	**0.089**
Grittiness	Mean	6.02	0.66	−14.06	−5.35	−20.08
	95% CI	(−12.66, 24.69)	(−16.81, 18.14)	(−32.77, 4.66)	(−25.09, 14.38)	(−38.88, −1.28)
	*P* value†				**0.589**	**0.037**

CI = confidence interval; FD hPL = fibrinogen-depleted human platelet lysate; N = number of patients in a specific group; VAS = Visual Analog Scale.

Baseline is defined as the last available value prior to the administration of the study drug.

The means, mean differences, confidence intervals, and *P* values are from a repeated measures model with treatment group, baseline severity category, visit, and treatment-by-visit interaction as fixed categorical effects using an unstructured covariance matrix.

The confidence intervals and *P* values are for mean change from baseline in comparison to the vehicle. *P* values are bolded.

† *P* values are 2-sided.

**Table 5:** Signs and Symptoms of Dry Eye: Comparison of Change from Baseline to Day 42 Between Treatments in the Double-Masked Treatment Phase

Parameter	Statistic	Vehicle Control (N = 22)	FD hPL 10% (N = 20)	FD hPL 30% (N = 22)	FD hPL 10%–Vehicle Control	FD hPL 30%-Vehicle Control
Tear film breakup time	Mean	−0.27	0.00	1.03	0.27	1.30
	95% CI	(−1.76, 1.23)	(−1.37, 1.37)	(−0.48, 2.55)	(−1.23, 1.77)	(−0.17, 2.77)
	*P* value†				**0.721**	**0.082**
Investigator’s global examination	Mean	4.52	3.47	3.66	−1.05	−0.86
	95% CI	(3.65, 5.39)	(2.66, 4.28)	(2.78, 4.54)	(−1.94, −0.16)	(−1.72, 0.01)
	*P* value†				**0.022**	**0.052**
Tear osmolarity	Mean	−10.83	−14.86	−19.71	−4.03	−8.88
	95% CI	(−22.22, 0.57)	(−25.32, −4.39)	(−31.28, −8.14)	(−15.32, 7.27)	(−19.98, 2.21)
	*P* value†				**0.478**	**0.114**
Ocular discomfort	Mean	−2.70	−11.44	−20.73	−8.74	−18.04
	95% CI	(−18.69, 13.29)	(−26.38, 3.51)	(−36.98, −4.49)	(−25.83, 8.36)	(−34.83, −1.25)
	*P* value†				**0.311**	**0.036**
OSDI	Mean	−10.83	−14.86	−19.71	−4.03	−8.88
	95% CI	(−22.22, 0.57)	(−25.32, −4.39)	(−31.28, −8.14)	(−15.32, 7.27)	(−19.98, 2.21)
	*P* value†				**0.478**	**0.114**
Lissamine green staining score	Mean	−2.11	−1.43	−1.29	0.67	0.82
	95% CI	(−5.5-, 1.29)	(−4.59, 1.72)	(−4.74, 2.16)	(−2.86, 4.21)	(−2.66, 4.29)
	*P* value†				**0.705**	**0.640**
Fluorescein sodium staining score	Mean	−0.34	−0.48	0.96	−0.14	1.30
	95% CI	(−1.58, 0.90)	(−1.65, 0.68)	(−0.30, 2.22)	(−1.48, 1.20)	(−0.01, 2.62)
	*P* value†				**0.833**	**0.052**
Schirmer’s Test	Mean	−0.19	1.24	−0.81	1.42	−0.62
	95% CI	(−2.30, 1.93)	(−0.75, 3.22)	(−2.96, 1.34)	(−0.85, 3.70)	(−2.86, 1.62)
	*P* value†				**0.216**	**0.581**

CI = confidence interval; FD hPL = fibrinogen-depleted human platelet lysate; N = number of patients in a specific group; OSDI = Ocular Surface Disease Index.

Baseline is defined as the last available value prior to the administration of the study drug.

The means, mean differences, confidence intervals, and *P* values are from a repeated measures model with treatment group, baseline severity category, visit, and treatment-by-visit interaction as fixed categorical effects using an unstructured covariance matrix.

The confidence intervals and *P* values are for mean change from baseline in comparison to the vehicle.

† *P* values are 2-sided and bolded.

Table 6 summarizes the percentages of patients with improvement in outcome measures by day 42 (double-masked treatment phase) or day 91 (open-label rollover phase) by treatment group. As seen in Table 6, fluorescein staining improved in the vehicle control and 10% groups and improved less in the 30% FD hPL group, a statistically significant difference. Lissamine green staining improved less in the 30% group than it did in the 10% FD hPL or control groups, though not significantly so. Schirmer testing of tear production did not show any significant change, consistent with prior autologous serum studies. Increased tear production is not felt to be a mechanism of action of this class of agents. Of the 22 patients in the control group, 17 subsequently entered the open-label rollover phase. In the open-label rollover phase, a mean decrease from baseline to day 91 (i.e., after 42 days of treatment with CAM-101 30%) in symptoms of burning/stinging of −22.0, eye discomfort of −27.9, eye dryness of −24.5, foreign body sensation of −1.3, grittiness of −19.5, itching of −25.3, pain of −22.1, and photophobia of −8.0 was seen. Like the original 30% treatment group, rollover patients had the greatest improvement in eye discomfort from baseline (day 42) to the end of treatment. Improvement was seen in > 50% of the rollover group for corneal staining, OSDI, global examination, eye discomfort, and all symptomatology except foreign body sensation.

**Table 6:** Percentage of Patients Improving on Day 42 (Double-Masked Treatment Phase) and Day 91 (Open-Label Rollover Phase), by Treatment Group

Parameter	Vehicle Control (N = 22) n (%)	Day 42 FD hPL 10% (N = 20) n (%)	Day 42 FD hPL 30% (N = 22) n (%)	Rollover (Day 91) FD hPL 30% (N = 17) n (%)
Corneal fluorescein stain	10 (45.5%)	12 (60.0%)	7 (31.8%)	10 (58.5%)
Corneal lissamine green	12 (54.5%)	13 (65.0%)	10 (45.5%)	8 (47.1%)
Total OSDI score	15 (68.2%)	16 (80.0%)	14 (63.6%)	10 (58.8%)
Tear osmolarity	5 (22.7%)	6 (30.0%)	7 (31.8%)	6 (35.3%)
Schirmer’s test	9 (40.9%)	9 (45.0%)	6 (27.3%)	7 (41.2%)
Investigator’s global examination	6 (27.3%)	13 (65.0%)	16 (72.7%)	11 (64.7%)
TBUT	12 (54.5%)	10 (50.0%)	13 (59.1%)	8 (47.1%)
Eye discomfort	13 (59.1%)	15 (75.0%)	17 (77.3%)	10 (58.8%)
Symptom frequency				
Burning/stinging	14 (63.6%)	10 (50.0%)	13 (59.1%)	11 (64.7%)
Itching	12 (54.5%)	10 (50.0%)	16 (72.7%)	10 (58.8%)
Foreign body	11 (50.0%)	15 (75.0%)	12 (54.5%)	7 (41.2%)
Eye discomfort	15 (68.2%)	14 (70.0%)	20 (90.9%)	13 (76.5%)
Eye dryness	11 (50.0%)	14 (70.0%)	15 (68.2%)	12 (70.6%)
Photophobia	10 (45.5%)	13 (65.0%)	15 (68.2%)	9 (52.9%)
Pain	13 (59.1%)	12 (60.0%)	13 (59.1%)	9 (52.9%)
Grittiness	12 (54.5%)	9 (45.0%)	14 (63.6%)	9 (52.9%)

For the double-masked phase, N = the number of patients randomized in a specific treatment group, and for the open-label phase, N = the number of patients planned to be enrolled in this group; n = number of patients with data available; calculation of percentages based on N; OSDI = Ocular Surface Disease Index; TBUT = tear breakup time.

## Discussion

This study was designed primarily to assess safety of FD hPL, and we observed no indication of toxicity for either the 10% or 30% FD hPL product. In addition to being well tolerated, we observed clinically and statistically significant improvement in most symptoms of dry eye among subjects treated with the 30% FD hPL product. We also observed a trend toward improvement in tear breakup time in the treatment groups. Of note, most subjects in the control group who elected to continue on study and receive treatment with 30% FD hPL experienced significant improvements in dry eye symptoms and in some dry eye signs. A potential explanation for the lack of improvements in some endpoints in the rollover group is worsening of signs or symptoms during an extended time on no therapy (42 days of Plasma-Lyte A vehicle) among subjects who had previously been on long-term ocular therapies. Another possibility is that FD hPL decreased corneal sensation, a parameter which we did not measure.

Lack of correlation between subjective and objective measures of tear deficiency is common in clinical trials and clinical practice.^16^, ^17^, ^18^ Although it could be argued that improvement in symptoms alone is an appropriate indication for dry eye treatment, improvement in objective tests, particularly staining evidence of ocular surface damage, would help better identify the mechanism(s) of action. It is possible that the measurements used in this study had low sensitivity or that the current study did not have the statistical power to detect changes in clinical signs. Corneal staining and conjunctival staining have been considered key measures of ocular surface damage. The Oxford Scale for fluorescein staining used in this trial, however, is dependent on the accuracy of each clinical site observer matching clinical findings to a series of standard panels and uses a relatively coarse gradation. Since most of the patients had severe DED at baseline, with high-grade fluorescein staining, the broad range of severity within each grade of fluorescein staining may have caused within-grade improvement to be missed by this measure, a limiting plateau effect. In understanding the mechanism of action of FD hPL and the currently accepted objective testing, the study treatment duration was likely also not long enough (6 weeks) to permit the product to potentially repair the corneal surface and normalize the staining. While the observed increase in fluorescein staining in the 30% group might indicate epithelial toxicity, this was not confirmed with the lissamine green stain, and fluorescein staining showed slight decreases in the control and 10% FD hPL groups. There were also limitations in how the sites performed and recorded data for some of the staining tests as well as tear osmolarity testing. Variations between study sites notwithstanding, subjects enrolled at a small subset of sites did show improvement in staining scores with 30% FD hPL. While we found no statistical evidence for corneal toxicity, longer treatment duration and objective and more granular measures of corneal staining would be useful in future trials.^19^ The trend toward improvement in tear breakup time with 30% FD hPL suggests an effect on meibomian gland function or some enhancement of epithelial cells. This effect might also be enhanced by a longer treatment period. Larger studies are required to confirm these effects.

There was slight improvement in the placebo group with respect to several of the outcome measures. Improvement in the placebo group may have occurred simply by chance, since signs and symptoms of tear deficiency are notoriously variable. Refresh Plus was also used during the 2-week washout period prior to baseline examinations, with patients randomized to the control group subsequently receiving Plasma-Lyte A for 6 weeks as their “study drug.” Improvements in the placebo group may thus have been attributable to the inherent properties of Plasma-Lyte A, which contains water and electrolytes, and its ability to hydrate the ocular surface. It may also have been a true placebo effect, which is typically seen in about 30% of placebo control populations in clinical studies (which might further explain the disconnection between subjective measures and objective measures). It may have resulted from discontinuation of harmful products prior to the run-in period, with 14 days being insufficient to see this effect. A longer run-in with more stringent exclusion of responders might mitigate this problem in future studies.

Limitations of this pilot study include the small sample size and short duration of treatment. The ranges of drug concentrations and frequency were narrow. The study population was heterogenous, and there was variability between study sites in staining classification.

Autologous serum tears have been shown to benefit patients with chronic ocular GvHD.^20^ Platelet-rich plasma has also been used to successfully treat dry eye ocular surface disorders.^21^, ^22^, ^23^ Autologous PRP lysate improved signs and symptoms in patients with GvHD who had not responded to conventional therapy^12^; Alio et al reported improvement in signs and symptoms of DED with topical PRP.^21^^,^^22^ Topical autologous PRP has also been shown to be safe and effective for the treatment of acute corneal chemical injuries and nonhealing sterile corneal ulcers.^24^

There are practical limitations to the use of autologous blood products as ocular therapies. These include the need for periodic blood draws, the lack of standardization in the preparation of ASTs and platelet-enriched plasma drops, the unknown shelf life of AST preparations, the common use of nonpreserved multidose packaging, and the practical difficulties patients face in storing these products frozen or refrigerated; all have hindered their widespread use for treating GvHD and other forms of severe tear deficiency. Additionally, serum and PRP drops from patients with significant systemic comorbidity and using systemic immunomodulating medications may negatively impact efficacy. Pooling of platelets from many healthy donors (allogeneic vs. autologous) may minimize batch-to-batch variations in concentrations of platelet-derived growth factors and cytokines that occur in individual patient blood draws, putatively providing the benefit of such products.^25^

## Conclusion

In this phase I/II pilot trial, FD hPL appears to be well tolerated, with no toxicities reported among the treatment groups. Consistent with a safety study, sample sizes were relatively small in a study population considered to have very severe DED. As a result, this study was not powered or intended to show statistically significant improvements in signs and symptoms. Nevertheless, these data do demonstrate numerous statistically significant improvements in outcome measures. These positive trends in this complex and severe test population suggest that a larger size and possibly longer study might document significant improvements in multiple additional outcome measures. Thus, FD hPL, a complex agent with multiple biologic components, shows promise in the treatment of multifactorial ocular surface diseases such as GvHD chronic dry eye. Further trials using 30% FD hPL in larger groups are planned to validate these findings.

## Supplementary Data

Collaborators
