# Supplementary material for: A Randomized Trial of Topical Fibrinogen-Depleted Human Platelet Lysate Treatment of Dry Eye Secondary to Chronic Graft-versus-Host Disease
Source: Ophthalmol Sci. 2022 Jun 2;2(3):100176. doi: 10.1016/j.xops.2022.100176 (PMC9562362; doi:10.1016/j.xops.2022.100176)
Supplement: Collaborators [file mmc1.docx]

Collaborators:

University of Michigan Kellogg Eye Center: Alan Sugar MD, Shahzad Mian MD, Roni Shtein MD, H. Kaz Soong MD, Munira Hussain. Oregon Health and Science University: Winston Chamberlain MD PhD, Afshan Nanji MD MPH, John Clements MD, Jennifer Maykovski, Paula Cisternas Labadzinzki. Massachusetts Eye and Ear Infirmary, Department of Ophthalmology, Harvard Medical School, Boston, MA: Reza Dana MD, Jia Jin MD, Joseph Ciolino MD, John Caccaviello. University of Washington: D. Patrick Kelly MD, Roya Habibi OD. Stanford University: Christopher Ta MD, Charles Yu MD, Charles Lin MD, Kristin Hirabayashi MD, Gabriel Valerio MD, Supriya Kawale, Mariana Nunez. Doheny Eye Center, University of California Los Angeles: John Irvine MD, Olivia Lee MD, Matthew Chu. Duke University Eye Center: Melissa Daluvoy MD, Victor Perez MD, Elmer Balajonda, Terry Hawks. University of Minnesota: Joshua Olson MD, Amanda Maltry MD, Joshua Hou MD, Wendy Elasky. University of Pittsburgh Eye Center: Vishal Jhanji MD, Rose Carla Aubourg. Woolfson Eye Institute and Cambium Medical Technologies LLC: R. Doyle Stulting MD PhD. Winship Cancer Institute of Emory University and Cambium Medical Technologies LLC: Edmund Waller MD PhD. Cambium Medical Technologies LLC: Neera Jagirdar MD, Terence Walts.
